# Localization of Neuronal Gain Control in the Pupillary Response

**DOI:** 10.3389/fneur.2019.00203

**Published:** 2019-03-12

**Authors:** Corinne Frances Carle, Andrew Charles James, Yanti Rosli, Ted Maddess

**Affiliations:** ^1^John Curtin School of Medical Research, Eccles Institute of Neuroscience, The Australian National University, Canberra, ACT, Australia; ^2^ANU Medical School, The Australian National University, Canberra, ACT, Australia; ^3^Diagnostic and Applied Health Sciences, Biomedical Science Program, Faculty of Health Science, Universiti Kebangsaan Malaysia, Kuala Lumpur, Malaysia

**Keywords:** pupil, gain-control, neural pathways, visual fields, perimetry, pupillometry, multifocal

## Abstract

Multifocal pupillographic objective perimetry (mfPOP) is being developed as an alternative to standard visual perimetry. In mfPOP, pupil responses to sparse multifocal luminance stimuli are extracted from the overall composite response. These individual test-region responses are subject to gain-control which is dependent on the temporal and spatial density of stimuli. This study aimed to localize this gain within the pupil pathway. Pupil constriction amplitudes of 8 subjects (41.5 ±12.7 y, 4 male) were measured using a series of 14 mfPOP stimulus variants. The temporal density of stimulus signal at the levels of retina, pretectal olivary nuclei (PON), and Edinger-Westphal nuclei (EWN) were controlled using a combination of manipulation of the mean interval between stimulus presentations (3 or 6 stimuli/s/hemiretina) and the restriction of stimuli to specific subsets of the 24 visual field test-regions per eye (left or right eye, left or right hemifield, or nasal or temporal hemifield). No significant difference was observed between mfPOP variants with differing signal density at the retina or PON but matched density at the other levels. In contrast, where signal density differed at the EWN but was the same at the retinal and PON levels e.g., between 3 stim/s *homonymous hemifield* and *all test-region* variants, significant reductions in constriction amplitudes were observed [*t*_(30)_ = −2.07 to −2.50, all *p* < 0.05]. Similar, although more variable, relationships were seen using nasal, and temporal hemifield stimuli. Results suggest that the majority of gain-control in the subcortical pupillary pathway occurs at the level of the EWN.

## Introduction

Far from being the product of a simple reflex arc, the pupillary luminance response has been shown to reflect quite complex processing of visual information. In addition to the diversity of signal arising from intrinsically photosensitive retinal ganglion cells (1–3) and various regions of visual cortex (4–7), non-linear gain-control acts within these pathways to modulate the size of the resulting pupillary constrictions. We have previously reported on the segregation and summation of pupillary visual signal (8) and have observed that constriction amplitudes are modulated on the basis of a combination of luminance intensity and temporal and spatial density of inputs (9–11). Presenting a number of stimuli simultaneously, or in close temporal proximity, to different areas of the visual-field does not produce a constriction that is equivalent to the product of the response to a single stimulus and the number of test-regions stimulated. The overall summed response is instead somewhat less than this, and therefore the shared response attributed to each of numerous stimuli will be less than that obtained to a single isolated stimulus. This is likely to be the effect of a divisive or subtractive gain mechanism (12) however the location of this neuronal gain-control within the pupillary pathway is at present unclear.

Localizing pupillary gain is an important goal because the pupil response is commonly used in the clinical detection and assessment of pathological conditions affecting both afferent and efferent pathways. It will allow for better interpretation of results and for tests to be designed that produce more accurate representations of function within different parts of the pupillary pathway. Our recent development of multifocal pupillographic objective perimetry (mfPOP) provides a unique means to achieve this aim. This technique measures the composite response of the pupils to sparse multifocal luminance stimuli that are presented concurrently to left and right eyes (13–15). In this study we aim to manipulate the temporal density of visual signal within the retina, PON, and Edinger-Westphal nuclei (EWN) using a combination of differences in the mean interval between stimulus presentations (either 3 or 6 stimuli/s/hemiretina) and by the restriction of stimuli to specific areas of the visual field (left or right eye, left or right hemifield, or nasal or temporal hemifield). Comparison between conditions in which the density of visual signal is the same with those in which it differs will therefore allow the assessment of gain at each of the levels of the pupillary pathway.

## Methods

### Subjects

Participants in this study were 8 subjects (4 male) with corrected to normal vision (mean age 41.5 ± 12.7 y). Each subject underwent testing with 14 different mfPOP variants across 5 sessions (2 or 3 variants per session) over a period of three days. Visual acuity was checked and visual fields were assessed using Humphrey FDT C-20 full threshold perimetry (Carl Zeiss Meditec, Inc., Dublin, CA, USA). Exclusion criteria included evidence of other ocular pathology or previous ocular surgery, refractive errors greater than ±6 diopters or more than 2 diopters of cylinder, or systemic disease or medication that might impair vision or pupillary responses. Subjects were requested not to consume caffeine or alcohol for 1 h before testing. Informed written consent was given by all participants after the nature and possible consequences of the study were explained, under ANU Human Experimentation Ethics Committee approval 238/04. All research adhered to the tenets of the Declaration of Helsinki.

### Multifocal Infrared Pupillography

**Figure 1:**
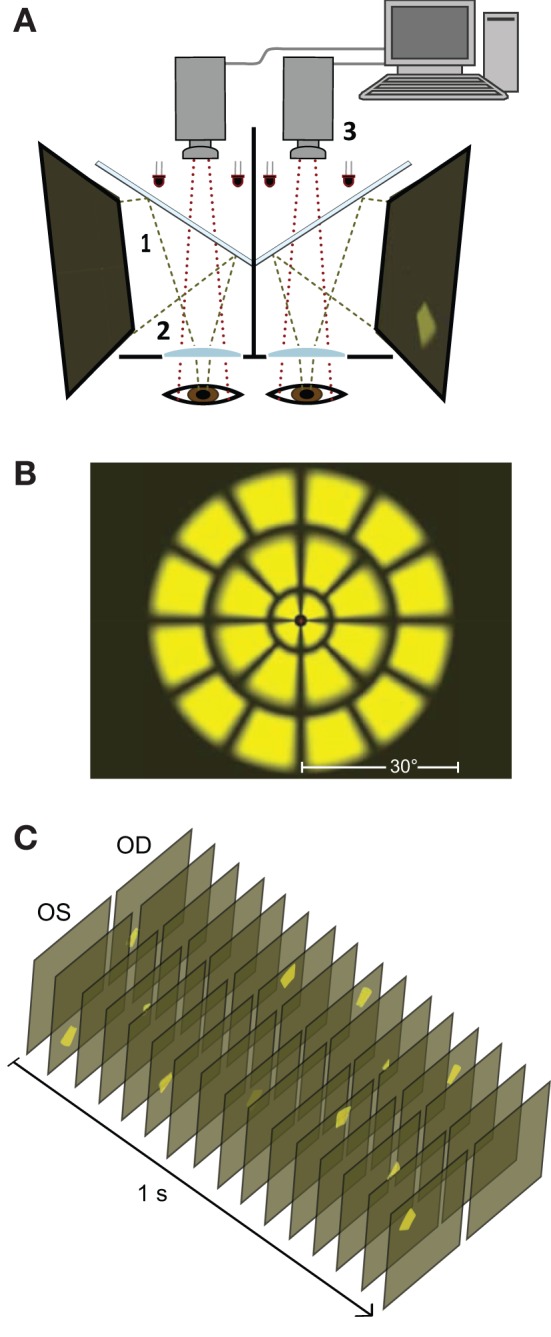
Stimulus presentation. **(A)** Stimuli are presented on two liquid crystal displays (LCDs, 1) with viewing distance set to optical infinity (2). Cold dichroic mirrors reflect the LCDs while allowing infrared light to illuminate subjects' eyes. Responses in both pupils are monitored using two infrared video cameras (3). **(B)** All the stimulus variants used in this study (Table 1) employed sub-sets of a basic 24 test-region per eye array. The array comprised 3 rings of stimuli extending ± 30° from fixation. **(C)** A 1 second duration representative excerpt of the stimulus sequence showing temporally and spatially sparse multifocal presentation for left (OS) and right (OD) eyes. This sample is consistent with the All test-regions both eyes 3 stimuli/s/hemiretina stimulus variant (Table 1, top row).

Presentation of stimuli and monitoring of pupil diameter were carried out using a prototype of the objectiveFIELD Analyzer (Konan Medical Inc., Irvine, CA, USA). This device uses concurrent, dichoptic presentation of sparse multifocal stimuli at 60 frames/s (9, 11, 16, 17). Infrared light is used to illuminate subjects' eyes and their pupillary responses are video monitored at 30 frames/s/eye (Figure 1A). Stimuli are presented at optical infinity to minimize accommodative responses. During testing, subjects fixated a small cross in the center of the viewing field. Binocular fusion of the two images was aided by large crosshairs. Gaze was monitored online, and data during blinks and fixation losses were deleted. Corrective lenses compensated for refractive errors to within 1.5 diopters; the stimuli contained no spatial frequencies above 2 cycles/degree, making them tolerant of this degree of misrefraction (18).

Processing of pupillary signal utilized custom-designed software developed using Matlab (release R2016b; MathWorks Inc., Natick, MA, USA). Response waveforms for each test-region were extracted from raw pupillary responses using multiple linear regression as previously described (19, 20). This method provided a set of 96 response estimates (waveforms) for each subject and stimulus variant i.e., direct and consensual responses for left and right eyes for each of the 24 test-regions. Thus, for each test-region, these response estimates are effectively the mean of the responses to either 60 or 120 individual stimulus presentations to that region, depending on the temporal density of the stimuli. As in previous studies, pupil measurements were normalized to a baseline pupil diameter of 3,500 μm (the estimated population mean) and are referred to as **AmpStd**. This provides constriction amplitudes for each eye of each subject that are relative to that standard diameter i.e., AmpStd = constriction amplitude ^*^(3,500/c) where c is the mean value of a trend line through the baseline pupil diameter record for each eye of each subject (9, 11, 15, 16).

### Stimuli

Stimulus layouts were based on a 24 region dartboard layout extending ±30° from fixation (Figure 1B). Yellow luminance-balanced stimuli of 33 ms duration were displayed on a 10 cd/m^2^ background at a maximum luminance of 150 cd/m^2^. Luminance-balancing involves lowering the luminance of stimuli relative to the inherent sensitivity of that test-region. This reduces topographic variation in constriction amplitudes and increases overall signal quality (21, 22). Multifocal stimulus presentation in this experiment was spatially as well as temporally sparse (Figure 1C) in contrast to the newer Clustered Volleys method (17, 19, 20). The mfPOP tests were of 4 min duration, broken into eight 30 s segments separated by short breaks.

**Figure 2:**
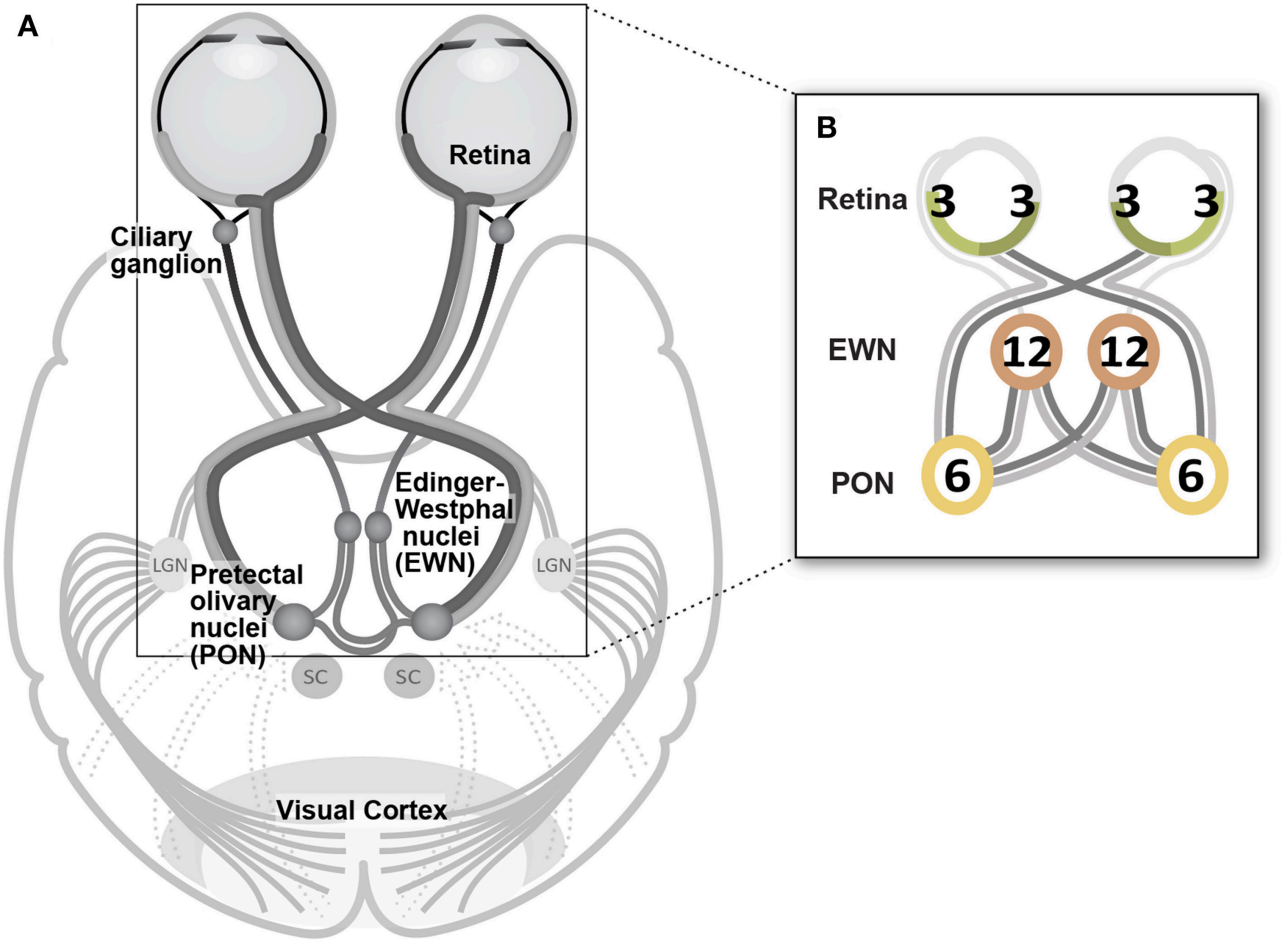
**(A)** Decussating and non-decussating input from retinal neurons arrives at the pretectal olivary nucleus (PON) via the brachium of the superior colliculus (SC) as well as from various regions of visual cortex. Axons of PON neurons project bilaterally to left and right Edinger-Westphal nuclei (EWN) forming a second hemi-decussation in the pathway. Parasympathetic fibers originating in the EWN travel within the inferior branch of the oculomotor nerve to the ciliary ganglion from where the fibers of the short ciliary nerves project to innervate the pupil constrictor muscle. **(B)** In the Results section, the temporal density of the luminance signal is indicated at each synapse in the pathway as shown in this example. Numerals reflect the amount of input arriving at each nucleus as the sum of the average total stimuli presented to each individual hemiretina (nasal or temporal) during a one second interval. Color coding reflects this: signal from 3 stimuli s^−1^ is shown in green, 6 stimuli s^−1^ hemiretina^−1^ in yellow, and 12 stimuli s^−1^ in orange. These signal densities are manipulated in the different experimental conditions (Table 1). As shown in this cartoon, lighter and darker hemiretinas, and projections as far as the EWN indicate the presence of temporal and nasal retinal signal, respectively, for each specific experimental condition.

The fourteen stimulus variants differed by the specific eye or visual hemifield in which stimuli were presented, as well as in the presentation rate of stimuli (Table 1, Figure 1C). Because ganglion cell axons from nasal and temporal retina follow different trajectories at the optic chiasm, the summed luminance signal arriving at each PON, and subsequently each EWN, will differ depending on the hemiretina of origin (Figure 2A). Comparisons were made between stimulus variants with estimates of signal density by linear summation as shown in Figure 2B. For example, compare a variant with stimuli presented in both hemifields of a single eye with another having stimuli restricted to homonymous hemifields of both eyes: both variants will have the same signal density within each stimulated hemiretina as well as at the EWN (which receives projections from both PON), but the second variant will have twice the signal density at the PON due to hemidecussation at the chiasm (this example is illustrated in **Figure 5B**). Using the differences in summed signal density between stimulus variants (Table 1), it is possible to construct comparisons such as this for retina, PON and EWN. Specific comparisons used will be described in the Results. The presentation rate condition, stimuli/s/hemiretina will be abbreviated to ***n/s/hr*** in the text, with *n* representing the number of stimuli presented.

**Table 1:** Stimulus protocol parameters for the 14 variants used in this study.

**Active** **test-regions**	**Presentation rate (stimuli/s/hemiretina)**	**Summed signal at each PON/s**	**Summed signal at each EWN/s**
All test-regions both eyes	3	6	12
	6	12	24
Left or right eye only^*^	3	3	6
	6	6	12
Left or right	3	6	6
homonymous hemifields^*^	6	12	12
Bitemporal hemifields	3	3	6
	6	6	12
Binasal hemifields	3	3	6
	6	6	12

*Subjects were tested with two protocols, at presentation rates of either 3 or 6 stimuli/second/hemiretina, for each of the active test-region variants*.

* *These active test-region variants had separate versions for each eye or hemifield i.e., left and right versions at each presentation rate, making four stimulus protocols each*.

### Data Analysis

Data analysis was undertaken using Matlab (R2016b; MathWorks Inc., Natick, MA, USA). Summary statistics are presented as the AmpStd median and median absolute deviation in the figures. Linear models were used for parametric testing of differences in constriction amplitudes between stimulus variants. In these regression models, the distribution of variance in responses was stabilized using a generalized logarithmic transform using a lambda (λ) value of 6 as described previously (14). Inputs to these regression models were based on the conservative assumption of complete within-subjects correlation. Thus, regressions were performed on the mean amplitudes across pupils, test-regions and, in the All Regions condition, eyes. This dataset was not large enough to accurately fit effects for factors such as sex or age so these were not included in the analysis.

To enable placement of specific comparisons within the overall dataset, the median amplitudes in **Figures 5,6** are presented in the context of the data from comparable conditions. The data pertaining to the comparisons illustrated in the accompanying cartoons are highlighted by a gray bar. For example in **Figure 5A** the comparison is between the 3/s/hr All Regions condition and the 6/s/hr Left and Right Eye conditions. The subsets of these data that were entered into the regression models are indicated by individual brackets for each comparison.

## Results

**Figure 3:**
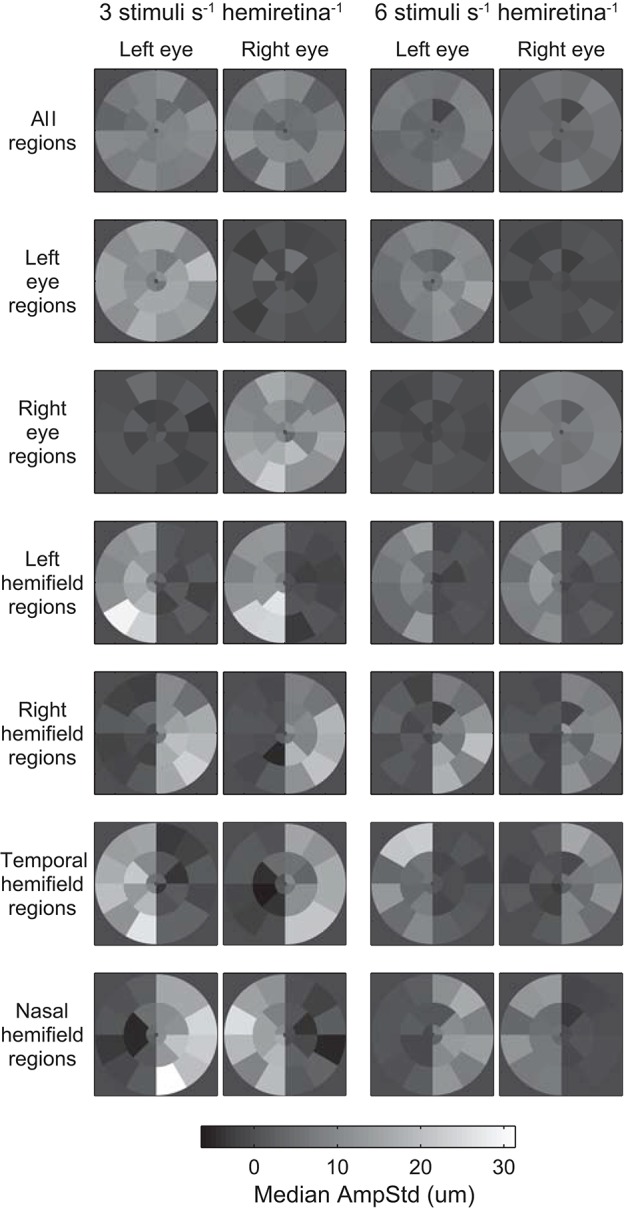
Median standardized constriction amplitudes (AmpStd) across pupils and subjects for each test-region and eye of each of the 14 stimulus variants. The rows of Table 1 correspond to the rows here.

**Figure 4:**
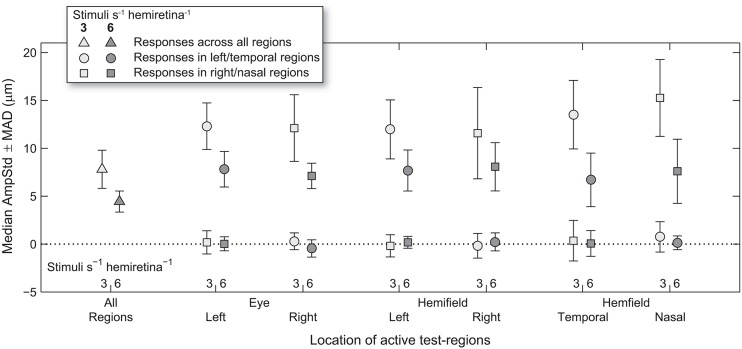
Median and median-absolute-deviation (MAD) of standardized constriction amplitudes (AmpStd) for the active and inactive subsets of test-regions in each stimulus variant across pupils, eyes (where relevant), and test-regions of all subjects.

Median test-region amplitudes across pupils and subjects ranged from below zero in regions in which stimuli were not presented to 31.4 μm AmpStd in stimulated regions (Figure 3). Within stimulated regions, constriction amplitudes varied according to the total number of regions stimulated as well as the temporal density of stimuli. These patterns can perhaps be seen more clearly in Figure 4, in which an overview of the medians across subsets of test-regions is presented. The median AmpStd for subsets where no stimuli were presented, e.g., right eye regions in variants where stimuli were presented to the left eye only, are close to zero in all instances. Of the region subsets where stimuli *were* presented, variants with a stimulus presentation rate of 6/s/hr produced smaller median amplitudes than the equivalent 3/s/hr variants in all cases. This is the expected outcome given the higher temporal density, and therefore larger summed signal and lower response gain, of the 6/s/hr variants. Selected data from this overview will be used to facilitate the comparison of constriction amplitudes under conditions of differing signal density at the retina, PON, and EWN.

### Retina

**Figure 5:**
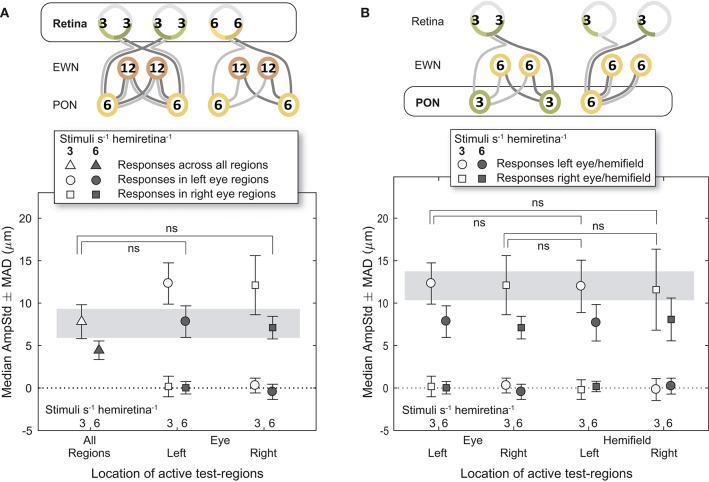
Effect of different stimulus presentation rates at the retinal and PON levels. In the cartoons at top, numerals represent the estimated summed signal density at each level of the pathway and the black outline the level of the pathway where the signal density differs between conditions. The gray bars indicate the data points relevant to these comparisons. **(A)** Doubling the signal density at the retina, while holding it the same in the pretectal olivary nuclei (PON) and Edinger-Westphal nuclei (EWN), did not significantly affect constriction amplitudes (AmpStd). **(B)** Doubling the signal density at the PON, while holding it the same in the retina and Edinger-Westphal nuclei (EWN), also did not significantly affect constriction amplitudes (AmpStd). “ns,” comparison not significant: *p* > 0.05.

Analysis of constriction amplitudes for variants in which the signal density differed at the level of the retina comprised a comparison between the 3/s/hr All Regions variant and the two 6/s/hr single eye variants (Figure 5A). Note that although the retinal signal density differs (3/s/hr vs. 6/s/hr), the summed signal at the PON (6/s/hr) and EWN (12/s/hr) are the same for each of these three variants. There was very little difference between constriction amplitudes for the All Regions variant and either of the Left or Right Eye variants; the small differences that were present were found to be non-significant [*t*_(21)_ = 0.22, *p* = 0.83, *t*_(21)_ = 0.46, *p* = 0.65, respectively]. This suggests that increasing the pooled signal density at the retinal level alone is unlikely to have any effect on constriction amplitudes.

### Pretectal Olivary Nuclei

The first comparison where signal density was manipulated to differ at the PON utilized 3/s/hr Left Eye (LE) and Right Eye (RE) variants contrasted against 3/s/hr Left and Right Homonymous Hemifield (LHH, RHH) variants (Figure 5B). In this comparison retinal (3/s/hr) and EWN (6/s/hr) signal are the same, but the signal density varies at the PON (3/s/hr vs. 6/s/hr). Amplitudes were very similar with none found to be significantly different (LE vs. LHH: *t*_(14)_ = 0.27, *p* = 0.79, LE vs. RHH: *t*_(14)_ = 0.03, *p* = 0.998, RE vs. LHH: *t*_(14)_ = 0.35, *p* = 0.73, RE vs. RHH: *t*_(14)_ = 0.10, *p* = 0.92). These results suggest that, as with the retina, doubling the signal density at the PON does not affect constriction amplitudes.

### Edinger-Westphal Nuclei

**Figure 6:**
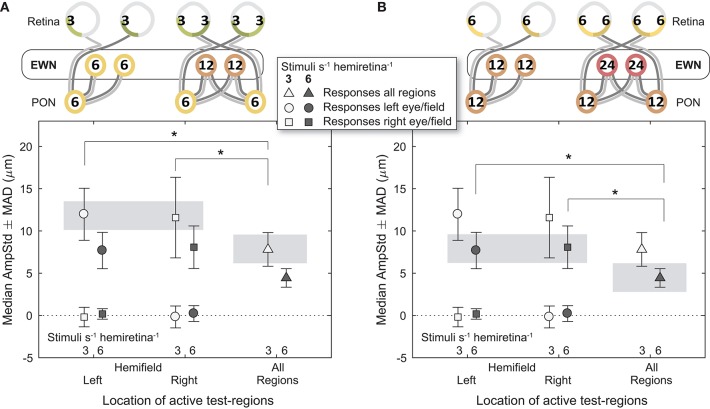
Effect of differing summed signal density at the Edinger-Westphal nuclei (EWN). In the cartoons at top, numerals represent the estimated summed signal density at each level of the pathway and the black outline the level of the pathway where the signal density differs between conditions. The gray bars indicate the data points relevant to these comparisons. **(A)** Increasing the signal density at the EWN, while holding it the same in the retina and pretectal olivary nuclei (PON), significantly reduced constriction amplitudes (AmpStd) in the 3/s/hr variants. **(B)** The 6/s/hr variants produced the same outcome, with significant differences in amplitude due to differing signal density at the EWN. ^*^p < 0.05.

Comparisons between different signal densities at the EWN firstly involved the 3/s/hr LHH and RHH variants, contrasted against the 3/s/hr All Regions variant (Figure 6A). Here, retinal (3/s/hr) and PON (6/s/hr) signal density were the same, and the EWN differed (6/s/hr vs. 12/s/hr). In contrast to the earlier results, the mean amplitude of the variant with the higher EWN signal density (3/s/hr All Regions) was significantly smaller than that of each of the other two variants [*t*_(30)_ = −2.26, *p* < 0.05, *t*_(30)_ = −2.28, *p* < 0.05). A similar comparison (Figure 6B) for the 6/s/hr variants also produced significant differences (*t*_(30)_ = −2.50, *p* < 0.05, *t*_(30)_ = −2.07, *p* < 0.05).

**Figure 7:**
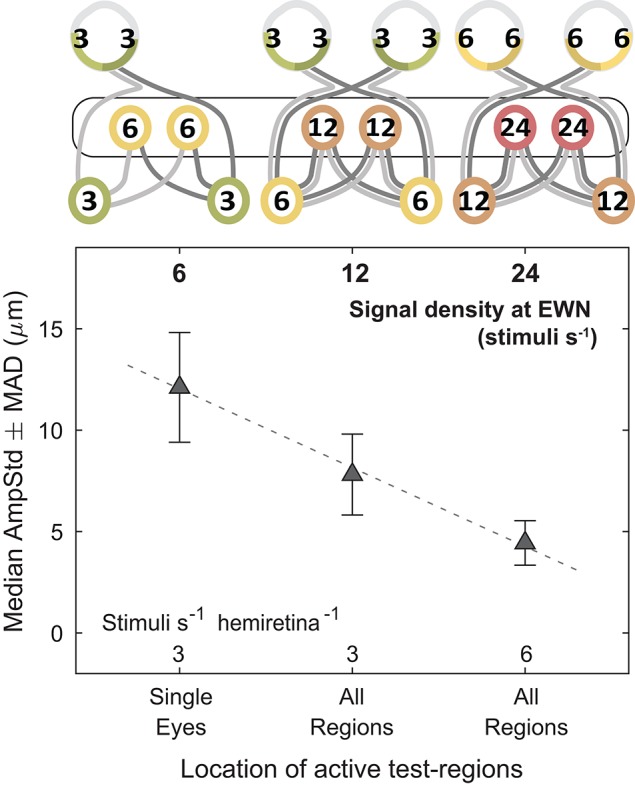
Effect of increasing the summed signal density at the Edinger-Westphal nuclei (EWN). In the cartoons at top, numerals represent the estimated summed signal density at each level of the pathway and the black outline the level of the pathway where the signal density differs between conditions. Doubling the summed signal density, equivalent to logarithmic increments of 0.301, resulted in a linear decrease in median pupil constriction amplitudes (AmpStd).

These results lend support to the hypothesis that modulation of pupillary responses due to changes in summed signal density occurs at the level of the EWN or later. As shown in Figure 7, progressively doubling the density of the visual signal at the EWN results in a linear decrease in constriction amplitudes within the range tested.

### Nasal and Temporal Hemifields

**Figure 8:**
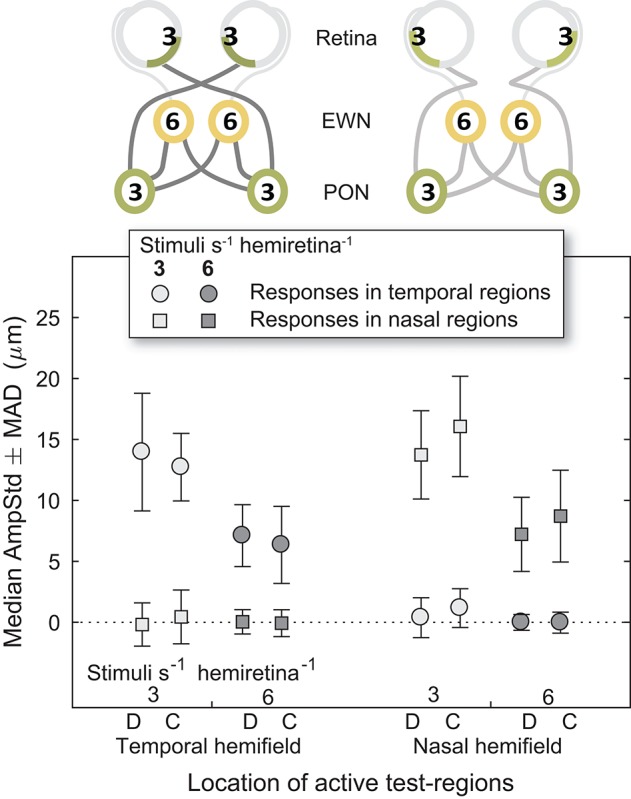
Direct and consensual responses for stimulus protocols with active test-regions in temporal or nasal hemifields. (On the abscissa, the letter D is used to indicate direct responses, C indicates consensual). In the cartoons at top, numerals represent the estimated summed signal density at each level of the pathway. Amplitudes for both 3/s/hr and 6/s/hr variants are plotted although only the 3/s/hr variants are represented in the cartoons.

Looking back at Figure 4, it can be seen that the regular pattern that has been seen so far does not appear to extend to variants in which Temporal and Nasal hemifield test-regions were stimulated in isolation from the opposite hemifield. The more sparse 3/s/hr condition responses are slightly larger than homonymous hemifield or individual eye variants, and the 6/s/hr conditions slightly smaller. In order to gain some insight into this irregularity, the medians of direct and consensual responses were estimated separately (Figure 8). Although not significant, consistent patterns emerged. On stimulation of the temporal hemifield, consensual responses appeared slightly smaller than direct. The opposite pattern occurred in the nasal hemifield. Although these results reveal at most a small trend, the direction of this trend is consistent with observations in the literature (23, 24). Further investigations targeting this specifically may yield useful information regarding the nature of the pooling of retinal signal.

## Discussion

In multifocal testing, many stimuli are presented in close temporal proximity. This means that the overall pupillary response at any given time will comprise temporally overlapping response components from a number of individual stimuli at different visual-field locations. Individual responses therefore, reflect just a proportion of this overall response. The actual number of stimuli that are summed will depend on the temporal density of the stimuli i.e., the stimulus presentation rate, as well as the time-constant, or memory, of the system. In this experiment, where very few stimuli were presented simultaneously (Figure 1C), it is clear that the gain of the system incorporates a temporal component since smaller amplitudes are obtained to the higher density 6/s/hr stimuli than 3/s/hr in all variants. Determining the location of this gain-control process within the pupillary pathway was the overarching aim of this project.

### Gain-Control Occurs in the Edinger-Westphal Nuclei

Responses were compared between stimulus variants having differing gain-states at a specific level of the pathway (retina, PON, EWN) while other levels of the system were subjected to equivalent gain. This experiment produced strong evidence for the Edinger-Westphal nuclei being the location of this gain-control mechanism, and no evidence of gain occurring at the retina or PON. This outcome may seem incongruous with the presence of GABAergic neurons in the rat PON (25) and the considerable degree of pooling of retinal inputs that occurs in the PON of primates (26); these two findings could point to the PON as a possible location for pupillary gain. PON luminance neuron outputs however, more closely resemble retinal signal than the pupillary response (27), leading to the alternative proposal that the EWN is the site of this signal modulation. Our results lend support to this latter hypothesis.

### Binocular vs. Monocular Summation

It is interesting to note that no difference was observed between summation of retinal signal across the two eyes and within the retina of a single eye (e.g., Figure 5B). Thompson in 1947 (28) reported that the area of monocular stimulation required to produce an equivalent constriction was four times that of the same area stimulated binocularly. There is no sign of this binocular amplification occurring in this study: our findings were more in line with those of Clarke et al. (29) in which binocular responses were slightly less than double the size of responses to otherwise identical monocular stimulation.

### Models of Gain and Integrity of Signal Within Pathways

It would appear from our results that pretectal large field luminance neurons likely pool information from the retina rather than modulate it, although the possibility exists that GABAergic PON neurons utilize a different time constant and e.g., may only modulate concurrent inputs. Varju (30) proposed a model of shunting inhibition for binocular summation but could only speculate as to where this might occur. His model proposes that the input from each retina is reduced proportionally by increases in input from the other i.e., maximal response is only obtained from stimulation of one retina when the other is in the dark. From our results it would seem likely that this same pattern may also apply for signal originating within the same eye. This point raises the question of how far along the pupillary pathway the retinotopy of signal is maintained. Our proposed model for signal summation and segregation in contraction anisocoria (8) would suggest that, at the least, pooling appears to maintain the separation of signal originating in different hemifields and eyes.

These experiments may have been somewhat limited by their use of an older version of mfPOP stimulus presentation than is currently used in our research (17); this is reflected by the relatively small stimulus amplitudes and variability of the results. The finding that constriction amplitudes were linear with the log of the stimulus presentation rate however, is consistent with Atchison's observations (31) that stimulus area is the reciprocal of luminance, given that pupil constrictions increase linearly with the log of the stimulus luminance over much of their range. Of course, these results do not preclude the existence of gain-control in other locations such as retinal adaptation of photoreceptors (32). The relatively small number of subjects in this study unfortunately prevented any exploration of variation in gain with age or across different populations. The localization of this gain-control to the EWN however, will act to inform the development of future pupillary stimuli and multifocal response extraction methods therefore leading to more accurate and reliable perimetric assessments. The findings also provide a starting point for further investigations into the precise nature of the pooling, segregation, and modulation of retinal signal within this nucleus, and broaden the knowledge surrounding the complexities of the pupillary response in humans.

## Data Availability

The raw data supporting the conclusions of this manuscript will be made available by the authors, without undue reservation, to any qualified researcher.

## Author Contributions

CC designed the study, undertook data acquisition and processing, designed and created the stimulus variants, analyzed the data and wrote the manuscript. AJ created the software for signal processing and stimulus generation. YR undertook data acquisition. TM provided scientific advice and oversight of the study and revised the manuscript.

### Conflict of Interest Statement

CC, AJ, and TM could potentially receive royalty income from patents assigned to Konan Medical USA Inc. The funder played no role in the study design, the collection, analysis or interpretation of data, the writing of this paper or the decision to submit it for publication. The remaining author declares that the research was conducted in the absence of any commercial or financial relationships that could be construed as a potential conflict of interest.

## References

[1] Gamlin PD McDougal DH Pokorny J Smith VC Yau KW Dacey DM. Human and macaque pupil responses driven by melanopsin-containing retinal ganglion cells. Vision Res. (2007) 47:946–54. 10.1016/j.visres.2006.12.015 17320141 PMC1945238

[2] Dacey DM Liao HW Peterson BB Robinson FR Smith VC Pokorny J. Melanopsin-expressing ganglion cells in primate retina signal colour and irradiance and project to the LGN. Nature. (2005) 433:749–54. 10.1038/nature03387 15716953

[3] McDougal DH Gamlin PD. The influence of intrinsically-photosensitive retinal ganglion cells on the spectral sensitivity and response dynamics of the human pupillary light reflex. Vision Res. (2010) 50:72–87. 10.1016/j.visres.2009.10.012 19850061 PMC2795133

[4] Barbur JL Harlow AJ Sahraie A Pupillary responses to stimulus structure, colour and movement. Opthalmic Physiol Opt. (1992) 12:137–41. 10.1111/j.1475-1313.1992.tb00276.x 1408159

[5] Heywood CA Nicholas JJ LeMare C Cowey A. The effect of lesions to cortical areas V4 or AIT on pupillary responses to chromatic and achromatic stimuli in monkeys. Exp Brain Res. (1998) 122:475–80. 10.1007/s002210050536 9827867

[6] Sahraie A Barbur JL. Pupil response triggered by the onset of coherent motion. Graefes Arch Clin Exp Ophthalmol. (1997) 235:494–500. 10.1007/BF00947006 9285218

[7] Gamlin PD. The pretectum: connections and oculomotor-related roles. Prog Brain Res. (2006) 151:379–405. 10.1016/S0079-6123(05)51012-4 16221595

[8] Carle CF Maddess T James AC. Contraction anisocoria: segregation, summation, and saturation in the pupillary pathway. Invest Ophth Vis Sci. (2011) 52:2365–71. 10.1167/iovs.10-6335 21212190

[9] Maddess T Ho YL Wong SS Kolic M Goh XL Carle CF. Multifocal pupillographic perimetry with white and colored stimuli. J Glaucoma. (2011) 20:336–43. 10.1097/IJG.0b013e3181efb097 20717051

[10] Sabeti F James AC Maddess T. Spatial and temporal stimulus variants for multifocal pupillography of the central visual field. Vision Res. (2011) 51:303–10. 10.1016/j.visres.2010.10.015 20951157

[11] Carle CF James AC Kolic M Loh YW Maddess T. High-resolution multifocal pupillographic objective perimetry in glaucoma. Invest Ophthalmol Vis Sci. (2011) 52:604–10. 10.1167/iovs.10-5737 20881285

[12] Ayaz A Chance FS. Gain modulation of neuronal responses by subtractive and divisive mechanisms of inhibition. J Neurophysiol. (2009) 101:958–68. 10.1152/jn.90547.2008 19073814

[13] James AC Kolic M Bedford SM Maddess T. Stimulus parameters for multifocal pupillographic objective perimetry. J Glaucoma. (2012) 21:571–8. 10.1097/IJG.0b013e31821e8413 21623219

[14] Carle CF James AC Maddess T. The pupillary response to color and luminance variant multifocal stimuli. Invest Ophthalmol Vis Sci. (2013) 54:467–75. 10.1167/iovs.12-10829 23188728

[15] Maddess T Bedford SM Goh XL James AC. Multifocal pupillographic visual field testing in glaucoma. Clin Exp Ophthalmol. (2009) 37:678–86. 10.1111/j.1442-9071.2009.02107.x 19788664

[16] Bell A James AC Kolic M Essex RW Maddess T. Dichoptic multifocal pupillography reveals afferent visual field defects in early type 2 diabetes. Invest Ophthalmol Vis Sci. (2010) 51:602–8. 10.1167/iovs.09-3659 19643957

[17] Sabeti F Maddess T Essex RW Saikal A James A Carle C. Multifocal pupillography in early age-related macular degeneration. Optom Vis Sci. (2014) 91:904–15. 10.1097/OPX.0000000000000319 24987814

[18] Anderson AJ Johnson CA. Frequency-doubling technology perimetry and optical defocus. Invest Ophth Vis Sci. (2003) 44:4147–52. 10.1167/iovs.02-1076 12939339

[19] James AC. The pattern-pulse multifocal visual evoked potential. Invest Ophthalmic Visual Sci. (2003) 44:879–90. 10.1167/iovs.02-0608 12556425

[20] James AC Ruseckaite R Maddess T. Effect of temporal sparseness and dichoptic presentation on multifocal visual evoked potentials. Visual Neurosci. (2005) 22:45–54. 10.1017/S0952523805221053 15842740

[21] Carle CF James AC Kolic M Essex RW Maddess T. Blue multifocal pupillographic objective perimetry in glaucoma. Invest Ophthalmic Visual Sci. (2015) 56:6394–403. 10.1167/iovs.14-16029 26444720

[22] Carle CF James AC Kolic M Essex RW Maddess T. Luminance and colour variant pupil perimetry in glaucoma. Clin Exp Ophthalmol. (2014) 42:815–24. 10.1111/ceo.12346 24725214

[23] Cox TA Drewes CP. Contraction anisocoria resulting from half-field illumination. Am J Ophthalmol. (1984) 97:577–82. 10.1016/0002-9394(84)90375-1 6720835

[24] Martin TL Kardon R Thompson HS Unequal direct and consensual pupillary responses to hemiretinal stimuli. Invest Ophth Vis Sci. (1991) 32:1124.

[25] Campbell G Lieberman AR. The olivary pretectal nucleus: experimental anatomical studies in the rat. Philos Transac R Soc Lond B Biol Sci. (1985) 310:573. 10.1098/rstb.1985.0132 2865761

[26] Clarke RJ Zhang H Gamlin PD. Primate pupillary light reflex: receptive field characteristics of pretectal luminance neurons. J Neurophysiol. (2003) 89:3168–78. 10.1152/jn.01130.2002 12611972

[27] Pong M Fuchs AF. Characteristics of the pupillary light reflex in the macaque monkey: discharge patterns of pretectal neurons. J Neurophysiol. (2000) 84:964–74. 10.1152/jn.2000.84.2.964 10938320

[28] Thomson LC. Binocular summation within the nervous pathways of the pupillary light reflex. J Physiol. (1947) 106:59–65. 10.1113/jphysiol.1947.sp004192 16991741 PMC1393690

[29] Clarke RJ Zhang H Gamlin PD. Characteristics of the pupillary light reflex in the alert rhesus monkey. J Neurophysiol. (2003) 89:3179–89. 10.1152/jn.01131.2002 12611973

[30] Varju D Human pupil dynamics. In: Proceedings of the International School of Physics “Enrico Fermi”. New York, NY: Academic Press (1969). p. 442–64.

[31] Atchison DA Girgenti CC Campbell GM Dodds JP Byrnes TM Zele AJ. Influence of field size on pupil diameter under photopic and mesopic light levels. Clin Exp Optometry. (2011) 94:545–8. 10.1111/j.1444-0938.2011.00636.x 21929524

[32] Shapley R Enroth-Cugell C Bonds AB Kirby A. Gain control in the retina and retinal dynamics. Nature. (1972) 236:352. 10.1038/236352a0 4553648

